# Supplementary material for: Detection of PTCH1 Copy-Number Variants in Mosaic Basal Cell Nevus Syndrome
Source: Biomedicines. 2024 Jan 31;12(2):330. doi: 10.3390/biomedicines12020330 (PMC10886644; doi:10.3390/biomedicines12020330)
Supplement: Supplementary file 1 [file biomedicines-12-00330-s001.zip › FigureS1 PTCH1 CNV analysis by MLPA.pdf]

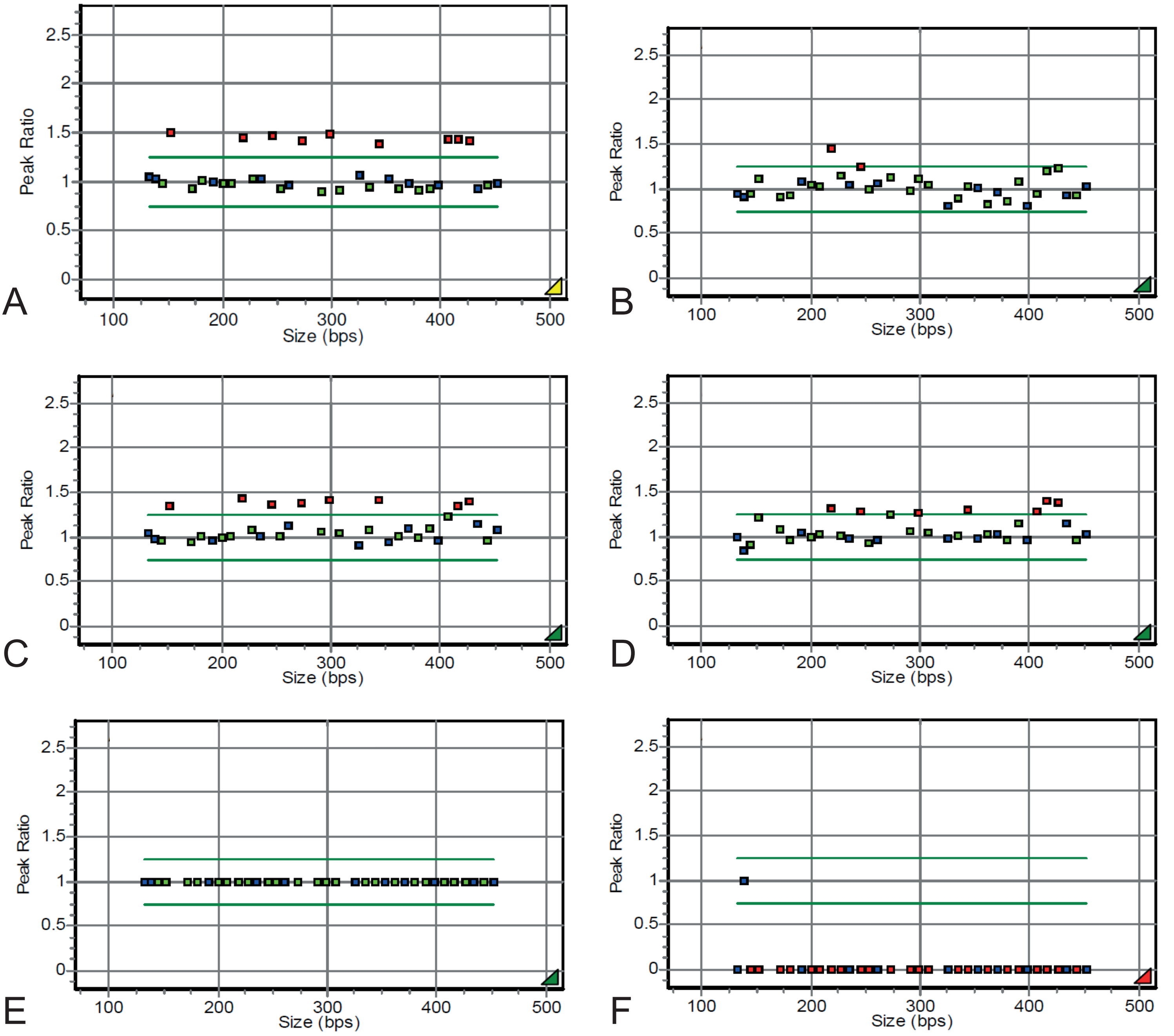

Figure S1 (A-F): *PTCH1* CNV analysis by MLPA based on DNA isolated from blood of the index patient (A) and blood (B) , hair (C) and saliva (D) of the index's father. The relative probe copy number ratio was calculated with respect to healthy control DNA (E) and a negative control was included (F). The red and green dots represent the copy number ratios (y-axis) of the 23 *PTCH1* probes located in each exon (except for exon 1 and 9), and the blue dots represent the reference probes. Copy number peak ratios are normalized to the reference probes and relative to the healthy control DNA sample. The green lines represent the cutoff values for significant copy number gain (red, >1.25) and copy number loss (<0.75).
