# Supplementary material for: Detection of PTCH1 Copy-Number Variants in Mosaic Basal Cell Nevus Syndrome
Source: Biomedicines. 2024 Jan 31;12(2):330. doi: 10.3390/biomedicines12020330 (PMC10886644; doi:10.3390/biomedicines12020330)
Supplement: Supplementary file 1 [file biomedicines-12-00330-s001.zip › FigureS2 Raw PTCH1 CNV MLPA analysis data.pdf]

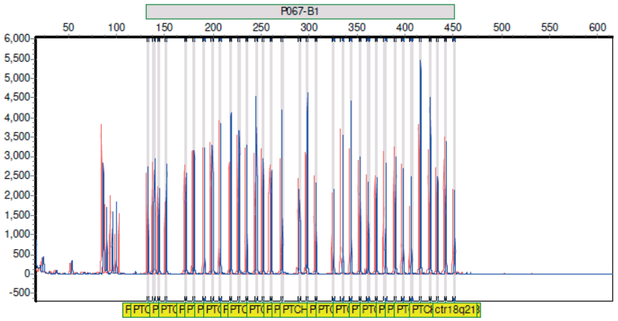

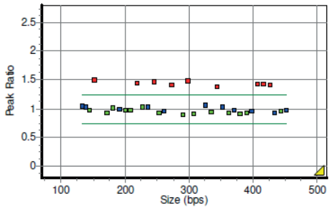

A

	Probe Name	Bin Size	21_E03_as_DNA15-61701
1	PTCH1 exon02	172.1	0.929
2	PTCH1 exon06	208.4	0.984
3	PTCH1 exon07	219.0	1.447
4	PTCH1 exon08	298.5	1.492
5	PTCH1 exon09	152.3	1.508
6	PTCH1 exon10	245.6	1.475
7	PTCH1 exon11	426.0	1.416
8	PTCH1 exon13	406.5	1.442
9	PTCH1 exon14	343.9	1.384
10	PTCH1 exon15	272.4	1.416
11	PTCH1 exon16	416.1	1.429
12	PTCH1 exon17	390.5	0.923
13	PTCH1 exon18	442.3	0.967
14	PTCH1 exon19	307.3	0.915
15	PTCH1 exon20	144.2	0.974
16	PTCH1 exon21	335.0	0.949
17	PTCH1 exon22	181.0	1.007
18	PTCH1 exon23	199.8	0.985
19	PTCH1 exon24	361.9	0.925
20	PTCH1 exon25	227.7	1.033
21	PTCH1 exon26	252.7	0.926
22	PTCH1 exon27	290.3	0.894
23	PTCH1 exon28	379.6	0.909
24	**ctr11q22**	370.4	0.978
25	**ctr16q22**	191.6	0.994
26	**ctr17p12**	433.9	0.921
27	**ctr18q21**	451.6	0.980
28	**ctr19p13**	325.9	1.056
29	**ctr1q23**	397.8	0.956
30	**ctr21q22**	132.9	1.051
31	**ctr2q13**	235.5	1.023
32	**ctr2q36**	260.7	0.956
33	**ctr7q31**	139.2	1.028
34	**ctr8q22**	353.2	1.027

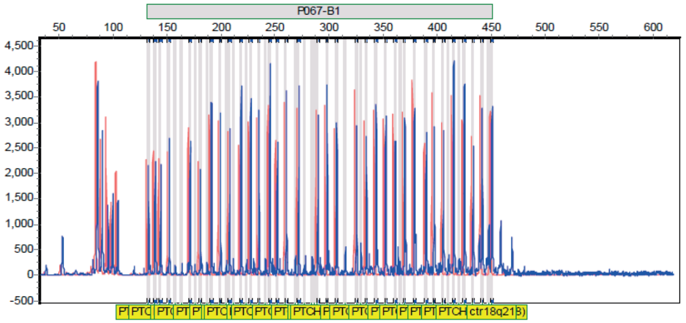

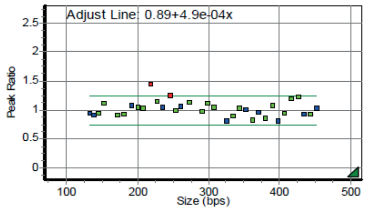

B

	Probe Name	Bin Size	04_D01_ast_repro_DNA15-74892
1	PTCH1 ex10 (13)	406.5	0.950
2	PTCH1 ex11 (14)	343.9	1.034
3	PTCH1 ex12 (15)	272.4	1.131
4	PTCH1 ex13 (16)	416.1	1.193
5	PTCH1 ex14 (17)	390.5	1.080
6	PTCH1 ex15 (18)	442.3	0.931
7	PTCH1 ex16 (19)	307.3	1.040
8	PTCH1 ex17(20)	144.2	0.944
9	PTCH1 ex18 (21)	335.0	0.898
10	PTCH1 ex19 (22)	181.0	0.929
11	PTCH1 ex20 (23)	199.8	1.047
12	PTCH1 ex21 (24)	361.9	0.834
13	PTCH1 ex22 (25)	227.7	1.154
14	PTCH1 ex23 (26)	252.7	0.991
15	PTCH1 ex24 (27)	290.3	0.974
16	PTCH1 ex25 (28)	379.6	0.854
17	PTCH1 ex3 (6)	208.4	1.022
18	PTCH1 ex4 (7)	219.0	1.452
19	PTCH1 ex5 (8)	298.5	1.117
20	PTCH1 ex6 (9)	152.1	1.114
21	PTCH1 ex7 (10)	245.6	1.251
22	PTCH1 ex8 (11)	426.0	1.232
23	PTCH1 exon02	171.9	0.904
24	**ctr11q22**	370.4	0.963
25	**ctr16q22**	191.4	1.080
26	**ctr17p12**	433.9	0.931
27	**ctr18q21**	451.6	1.029
28	**ctr19p13**	325.9	0.807
29	**ctr1q23**	397.8	0.813
30	**ctr21q22**	132.9	0.946
31	**ctr2q13**	235.5	1.049
32	**ctr2q36**	260.7	1.067
33	**ctr7q31**	139.2	0.916
34	**ctr8q22**	353.2	1.019

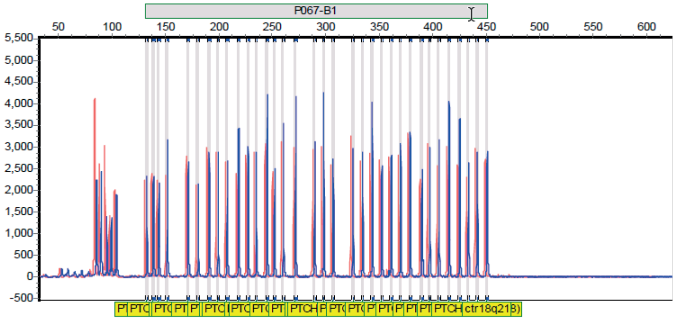

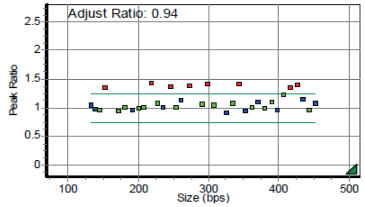

C

	Probe Name	Bin Size	01_A01_ast_repro_DNA16-65380
1	PTCH1 ex10 (13)	406.5	1.232
2	PTCH1 ex11 (14)	343.9	1.411
3	PTCH1 ex12 (15)	272.4	1.386
4	PTCH1 ex13 (16)	416.1	1.342
5	PTCH1 ex14 (17)	390.5	1.100
6	PTCH1 ex15 (18)	442.3	0.965
7	PTCH1 ex16 (19)	307.3	1.050
8	PTCH1 ex17(20)	144.2	0.969
9	PTCH1 ex18 (21)	335.0	1.074
10	PTCH1 ex19 (22)	181.0	1.006
11	PTCH1 ex20 (23)	199.8	0.998
12	PTCH1 ex21 (24)	361.9	1.013
13	PTCH1 ex22 (25)	227.7	1.076
14	PTCH1 ex23 (26)	252.7	1.021
15	PTCH1 ex24 (27)	290.3	1.060
16	PTCH1 ex25 (28)	379.6	1.002
17	PTCH1 ex3 (6)	208.4	1.011
18	PTCH1 ex4 (7)	219.0	1.429
19	PTCH1 ex5 (8)	298.5	1.414
20	PTCH1 ex6 (9)	152.1	1.350
21	PTCH1 ex7 (10)	245.6	1.365
22	PTCH1 ex8 (11)	426.0	1.404
23	PTCH1 exon02	171.9	0.951
24	**ctr11q22**	370.4	1.099
25	**ctr16q22**	191.4	0.963
26	**ctr17p12**	433.9	1.146
27	**ctr18q21**	451.6	1.073
28	**ctr19p13**	325.9	0.917
29	**ctr1q23**	397.8	0.968
30	**ctr21q22**	132.9	1.046
31	**ctr2q13**	235.5	1.007
32	**ctr2q36**	260.7	1.136
33	**ctr7q31**	139.2	0.979
34	**ctr8q22**	353.2	0.949

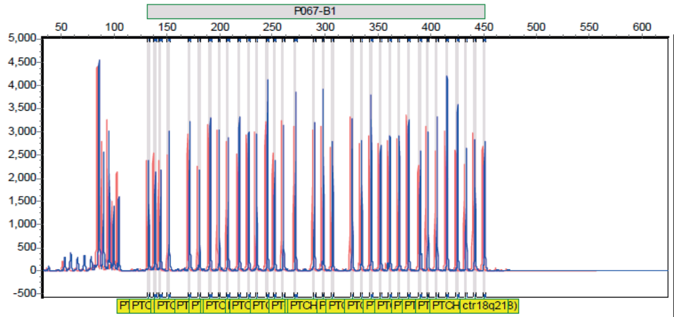

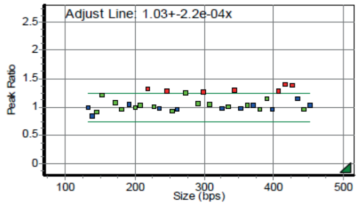

D

	Probe Name	Bin Size	02_B01_ast_repro_DNA16-65381
1	PTCH1 ex10 (13)	406.5	1.288
2	PTCH1 ex11 (14)	343.9	1.300
3	PTCH1 ex12 (15)	272.4	1.243
4	PTCH1 ex13 (16)	416.1	1.393
5	PTCH1 ex14 (17)	390.5	1.143
6	PTCH1 ex15 (18)	442.3	0.957
7	PTCH1 ex16 (19)	307.3	1.045
8	PTCH1 ex17(20)	144.2	0.908
9	PTCH1 ex18 (21)	335.0	1.021
10	PTCH1 ex19 (22)	181.0	0.965
11	PTCH1 ex20 (23)	199.8	1.002
12	PTCH1 ex21 (24)	361.9	1.035
13	PTCH1 ex22 (25)	227.7	1.020
14	PTCH1 ex23 (26)	252.7	0.937
15	PTCH1 ex24 (27)	290.3	1.059
16	PTCH1 ex25 (28)	379.6	0.970
17	PTCH1 ex3 (6)	208.4	1.030
18	PTCH1 ex4 (7)	219.0	1.318
19	PTCH1 ex5 (8)	298.5	1.261
20	PTCH1 ex6 (9)	152.1	1.209
21	PTCH1 ex7 (10)	245.6	1.281
22	PTCH1 ex8 (11)	426.0	1.379
23	PTCH1 exon02	171.9	1.089
24	**ctr11q22**	370.4	1.026
25	**ctr16q22**	191.4	1.044
26	**ctr17p12**	433.9	1.149
27	**ctr18q21**	451.6	1.036
28	**ctr19p13**	325.9	0.987
29	**ctr1q23**	397.8	0.965
30	**ctr21q22**	132.9	0.999
31	**ctr2q13**	235.5	0.987
32	**ctr2q36**	260.7	0.970
33	**ctr7q31**	139.2	0.841
34	**ctr8q22**	353.2	0.987

Figure S2(A-D): Raw *PTCH1* CNV MLPA analysis data based on DNA isolated from blood of the index patient (A) and blood (B), hair (C) and saliva (D) of the index's father. For each sample the fragment analysis electropherogram (upper left), the individual probe results including probe location, corresponding bin size and calculated ratio and the resulting peak ratio plot (See Supp. Figure S1) is displayed.
