# Supplementary material for: Detection of PTCH1 Copy-Number Variants in Mosaic Basal Cell Nevus Syndrome
Source: Biomedicines. 2024 Jan 31;12(2):330. doi: 10.3390/biomedicines12020330 (PMC10886644; doi:10.3390/biomedicines12020330)
Supplement: Supplementary file 1 [file biomedicines-12-00330-s001.zip › Table S1 Primer sequences used for PTCH1 Sanger sequencing.pdf]

Table S1: Primer sequences used for *PTCH1* Sanger sequencing.

ID	Sequence with M13	Primer sequence	Product size (bp)
**PTCH1_01_TR1_A_F**	**tgtaaaacgacggccagtTGAGTTTATTGTAAAGGGGTAAAGT**	**TGAGTTTATTGTAAAGGGGTAAAGT**	343
**PTCH1_01_TR1_A_R**	**caggaaacagctatgaccGGCTTGGATTTCACATCAATTC**	**GGCTTGGATTTCACATCAATTC**	343
**PTCH1_01_F**	**tgtaaaacgacggccagtCAGCAGCGTCCTCGCAA**	**CAGCAGCGTCCTCGCAA**	382
**PTCH1_01_R**	**caggaaacagctatgaccGGGGCGATCCCAAAGAG**	**GGGGCGATCCCAAAGAG**	382
**PTCH1_02_F**	**tgtaaaacgacggccagtCCCATGACGCTCAGATCC**	**CCCATGACGCTCAGATCC**	511
**PTCH1_02_R**	**caggaaacagctatgaccGCGCCCAAACAATAAACAAT**	**GCGCCCAAACAATAAACAAT**	511
**PTCH1_03_F**	**tgtaaaacgacggccagtTGCTCACACATCAGCCAGTCTCA**	**TGCTCACACATCAGCCAGTCTCA**	592
**PTCH1_03_R**	**caggaaacagctatgaccCCCAAGTGGCTCAGCGTCAT**	**CCCAAGTGGCTCAGCGTCAT**	592
**PTCH1_04_F**	**tgtaaaacgacggccagtCAGCTGGGCTGCCCTGAAGT**	**CAGCTGGGCTGCCCTGAAGT**	557
**PTCH1_04_R**	**caggaaacagctatgaccCCCTTCCCAGAAGCAGTCCAA**	**CCCTTCCCAGAAGCAGTCCAA**	557
**PTCH1_05_F**	**tgtaaaacgacggccagtGGCAGTGGAAATTGGAACATTTG**	**GGCAGTGGAAATTGGAACATTTG**	578
**PTCH1_05_R**	**caggaaacagctatgaccGCCTCGTCTTCGAAACTACGAACATT**	**GCCTCGTCTTCGAAACTACGAACATT**	578
**PTCH1_06_F**	**tgtaaaacgacggccagtTGCAGATATGCTGGAAAGGAGCA**	**TGCAGATATGCTGGAAAGGAGCA**	549
**PTCH1_06_R**	**caggaaacagctatgaccGGCTAATGGGAGGTGTATGGCA**	**GGCTAATGGGAGGTGTATGGCA**	549
**PTCH1_07_F**	**tgtaaaacgacggccagtCAATCCGGCCGATCCAGACT**	**CAATCCGGCCGATCCAGACT**	599
**PTCH1_07_R**	**caggaaacagctatgaccTGGCTAGCGAGGATAACGGTTT**	**TGGCTAGCGAGGATAACGGTTT**	599
**PTCH1_08_F**	**tgtaaaacgacggccagtGCGTCACTGAGCCCGGTGTAT**	**GCGTCACTGAGCCCGGTGTAT**	600
**PTCH1_08_R**	**caggaaacagctatgaccCCCATCAAGTTCCCAGAATTGC**	**CCCATCAAGTTCCCAGAATTGC**	600
**PTCH1_09_F**	**tgtaaaacgacggccagtACTCCAGCGACACCAGCCCT**	**ACTCCAGCGACACCAGCCCT**	567
**PTCH1_09_R**	**caggaaacagctatgaccAGGCAAACGGCAAATGGGAA**	**AGGCAAACGGCAAATGGGAA**	567
**PTCH1_10_F**	**tgtaaaacgacggccagtTTCCCATTTGCCGTTTGCCT**	**TTCCCATTTGCCGTTTGCCT**	530
**PTCH1_10_R**	**caggaaacagctatgaccTACCCACTTCCCTGAGGGCG**	**TACCCACTTCCCTGAGGGCG**	530
**PTCH1_11_F**	**tgtaaaacgacggccagtTGAGGTTACGGAAGCCCTGC**	**TGAGGTTACGGAAGCCCTGC**	430
**PTCH1_11_R**	**caggaaacagctatgaccCCGAGATGCAGCTCTTGGGA**	**CCGAGATGCAGCTCTTGGGA**	430
**PTCH1_12_F**	**tgtaaaacgacggccagtGGCAGGTGGCTCTGTTTCCC**	**GGCAGGTGGCTCTGTTTCCC**	558
**PTCH1_12_R**	**caggaaacagctatgaccCCCGAAAGCCATGCATAAAGG**	**CCCGAAAGCCATGCATAAAGG**	558
**PTCH1_13_F**	**tgtaaaacgacggccagtCCCAGGTTTGTGCCTTATCGG**	**CCCAGGTTTGTGCCTTATCGG**	521
**PTCH1_13_R**	**caggaaacagctatgaccGGGAAGCAGCCTCTGTCCAA**	**GGGAAGCAGCCTCTGTCCAA**	521
**PTCH1_14_A_F**	**tgtaaaacgacggccagtTCCTCCATGTGGCCTCCTCA**	**TCCTCCATGTGGCCTCCTCA**	544
**PTCH1_14_A_R**	**caggaaacagctatgaccCAGTGGAGGCTGGAGTCGGA**	**CAGTGGAGGCTGGAGTCGGA**	544
**PTCH1_14_B_F**	**tgtaaaacgacggccagtCCTCAGGCCTACACCGACACA**	**CCTCAGGCCTACACCGACACA**	457
**PTCH1_14_B_R**	**caggaaacagctatgaccCCTTTGGAGTTCATCAGATTGC**	**CCTTTGGAGTTCATCAGATTGC**	457
**PTCH1_15_F**	**tgtaaaacgacggccagtCCTTGGCAACACAAGACTGTCTCA**	**CCTTGGCAACACAAGACTGTCTCA**	593
**PTCH1_15_R**	**caggaaacagctatgaccCCTGTTGAAGCTGAACACGCAA**	**CCTGTTGAAGCTGAACACGCAA**	593
**PTCH1_16_F**	**tgtaaaacgacggccagtGAGCAGTGGCTGGCAGCAG**	**GAGCAGTGGCTGGCAGCAG**	593
**PTCH1_16_R**	**caggaaacagctatgaccTTCTGGTGGTAGGAACACGCC**	**TTCTGGTGGTAGGAACACGCC**	593
**PTCH1_17_F**	**tgtaaaacgacggccagtCGCCAGTGATTGCATCCTCC**	**CGCCAGTGATTGCATCCTCC**	582
**PTCH1_17_R**	**caggaaacagctatgaccTCCCAATGTGATAGAGTGCGGG**	**TCCCAATGTGATAGAGTGCGGG**	582
**PTCH1_18_F**	**tgtaaaacgacggccagtGCCTGGAGGCTATGATCAGCA**	**GCCTGGAGGCTATGATCAGCA**	588
**PTCH1_18_R**	**caggaaacagctatgaccCCATGGACCTCACCACCTCG**	**CCATGGACCTCACCACCTCG**	588
**PTCH1_19_F**	**tgtaaaacgacggccagtACAGTCTCCCTTCCCAGCGG**	**ACAGTCTCCCTTCCCAGCGG**	395

**PTCH1_19_R**	**caggaaacagctatgaccACTTGGAGACAAACAGAGCCAG**	**ACTTGGAGACAAACAGAGCCAG**	395
**PTCH1_20_F**	**tgtaaaacgacggccagtTCGGGGTGAGTATCAGTGAA**	**TCGGGGTGAGTATCAGTGAA**	663
**PTCH1_20_R**	**caggaaacagctatgaccCTCAGCCTCCCAAGTAGCTG**	**CTCAGCCTCCCAAGTAGCTG**	663
**PTCH1_21_F**	**tgtaaaacgacggccagtTTGAATGTGAACTGCGGTTGGA**	**TTGAATGTGAACTGCGGTTGGA**	430
**PTCH1_21_R**	**caggaaacagctatgaccGAACCCGCCCTCTAGCCCTC**	**GAACCCGCCCTCTAGCCCTC**	430
**PTCH1_22_F**	**tgtaaaacgacggccagtAATACGGCACAGTGCGCAGG**	**AATACGGCACAGTGCGCAGG**	590
**PTCH1_22_R**	**caggaaacagctatgaccTCAAACCACAGGAAGATGGCA**	**TCAAACCACAGGAAGATGGCA**	590
**PTCH1_23_A_F**	**tgtaaaacgacggccagtGCCCACATTTATGGGCAGCA**	**GCCCACATTTATGGGCAGCA**	600
**PTCH1_23_A_R**	**caggaaacagctatgaccGCGACAGTCACGGAGGCAGA**	**GCGACAGTCACGGAGGCAGA**	600
**PTCH1_23_B_F**	**tgtaaaacgacggccagtGGACGGCAAGGCCAGCAG**	**GGACGGCAAGGCCAGCAG**	584
**PTCH1_23_B_R**	**caggaaacagctatgaccGGGTCCAGCGTGGGATGTG**	**GGGTCCAGCGTGGGATGTG**	584
